# Supplementary material for: The structure and flexibility analysis of the Arabidopsis synaptotagmin 1 reveal the basis of its regulation at membrane contact sites
Source: Life Sci Alliance. 2021 Aug 18;4(10):e202101152. doi: 10.26508/lsa.202101152 (PMC8380656; doi:10.26508/lsa.202101152)
Supplement: Supplementary file 1 [file LSA-2021-01152_TableS1.docx]

**Table S1. Data collection and refinement statistics**

| Data collection |  |  |
| --- | --- | --- |
| Construct  Space group | SYT1C2A Ca  R 3 :H | SYT1C2A Cd  R 3 :H |
| a, b, c, Å  α, β, γ, °  Resolution, Å  Rpim  CC_1/2_  Ι/σ(Ι)  Completeness, %  Redundancy | 69.13 69.13 115.67  90 90 120  41.59-2.10 (2.16-2.10)  0.023 (0.706)  0.999 (0.551)  14.3 (1.1)  100.0 (100.0)  6.6 (6.8) | 70.94 70.94 116.31  90 90 120  42.23-2.00 (2.06-2.00)  0.053 (1.041)  0.997 (0.449)  7.6 (0.8)  99.5 (99.1)  3.4 (3.5) |
| Refinement |  |  |
| Resolution, Å  No. of reflections  Rwork/Rfree | 41.59-2.10 (2.18-2.10)  24010  20.7/26.9 (35.8/38.9) | 42.23-2.00 (2.16-2.00)  14573  18.6/21.1 (34.3/35.1) |
| No. of atoms |  |  |
| Protein  Calcium/Cadmium ions  Zinc/Nickel ions  Chlorine ions  Ethylene glycol  Water molecules  Average B factors | 1054  2  1  1  -  10  92.23 | 1074  2  2  1  2  28  62.98 |
| rmsd |  |  |
| Bond lengths, Å  Bond angles, º  Ramachandram plot | 0.009  0.971  96.0% in the core  4.0% in the allowed | 0.008  0.917  96.85% in the core  3.15% in the allowed |

Highest-resolution shell is shown in parenthesis
