# Supplementary material for: The structure and flexibility analysis of the Arabidopsis synaptotagmin 1 reveal the basis of its regulation at membrane contact sites
Source: Life Sci Alliance. 2021 Aug 18;4(10):e202101152. doi: 10.26508/lsa.202101152 (PMC8380656; doi:10.26508/lsa.202101152)
Supplement: Supplementary file 3 [file LSA-2021-01152_TableS2.docx]

**Table S2.** Data collection and structural parameters by SAXS.

|  |  | | | **C2AB** | | | **C2AB-Ca** | **SMPC2A(N34-E397)** | | |
| --- | --- | --- | --- | --- | --- | --- | --- | --- | --- | --- |
| **Data collection parameters** | |  | | | | | | | | |
| BeamLine | | B21, Diamond Light Source, Harwell (UK) | | | | | | | | |
| Detector | |  | | | Eiger 4M | | Eiger 4M | | | Pilatus 2M |
| Beam size (mm) | |  | | | 0.34 x 0.40 | | 0.34 x 0.40 | | | 0.2 x 0.2 |
| Energy (keV) | |  | | | 12.4 | | 12.4 | | | 12.4 |
| Sample-to-detector distance (mm) | |  | | 3700 | | | 3700 | | 4014 | |
| q range (A-1) | |  | | | 0.0026 – 0.34 | | 0.0026 – 0.34 | | | 0.0038 - 0.42 |
| Exposure time (s) | | 3 | | | | | | | | |
| Temperature (K) | | 293 | | | | | | | | |
| Data collection mode | | SEC online | | | | | | | | |
| **Structural parameters** | |  |  | | | | | |  | |
| Concentration range (mg ml^-1^) | |  | | 10 | | 10 | | 6.6 | | |
| *q* Interval for Fourier inversion (Å^-1^) | |  | | 0.011 – 0.258 | | 0.007 – 0.258 | | 0.010 -0.190 | | |
| *R_g_* [from P(r)] (Å) | |  | | 32.1 | | 30.6 | | 45.1 | | |
| *R_g_* [from Guiner approximation] (Å) | |  | | 28.2 | | 29.1 | | 44.9 | | |
| *sR_g_* limits [from Guiner approximation] | |  | | 0.33 1.30 | | 0.21 1.30 | | 0.41 1.28 | | |
| Dmax (Å) | |  | | 121 | | 114 | | 176 | | |
| Porod coefficient | |  | | 2.0 | | 2.1 | | 2.8 | | |
| Porod volume estimate (nm^3^) | |  | | 50 | | 54 | | 138 | | |
| DAMMIF excluded volume (nm^3^) | |  | | 55 | | 57 | | 151 | | |
| Molecular Mass (kDa) | |  | |  | |  | |  | | |
| From Porod volume (x0.53) | |  | | 28 | | 29 | | 73 | | |
| From excluded volume (x 0.5) | |  | | 28 | | 29 | | 76 | | |
| From sequence | |  | | 33 | | 33 | | 41 per protomer | | |
| Modelling | |  | |  | |  | |  | | |
| Ambiguity | |  | | 1.0 (is unique) | | 1.5 (might be ambiguous) | | 2.1 (might be ambiguous) | | |
| Resolution (Å) | |  | | 30 ± 2 | | 31 ± 3 | | 57 ± 4 | | |
| SASBDB | |  | SASDKJ9 | | | SASDKK6 | | SASDKG6 | | |
| **Software employed** | |  |  | | | | | |  | |
| Primary data reduction | |  | DAWN pipeline (Diamond Light Source, UK) | | | | | |  | |
| Data processing | |  | ScÅtter IV | | | | | |  | |
| Computation of model intensities | |  | CRYSOL | | | | | |  | |
| Flexibility | |  | MultiFoxs | | | | | |  | |
|  | |  |  | | | | | |  | |
|  | |  |  | | | | | |  | |

*q = 4πsin(θ)/λ*, where *2θ* is the scattering angle and *λ* is the wavelength od incident X ray beam
